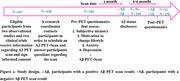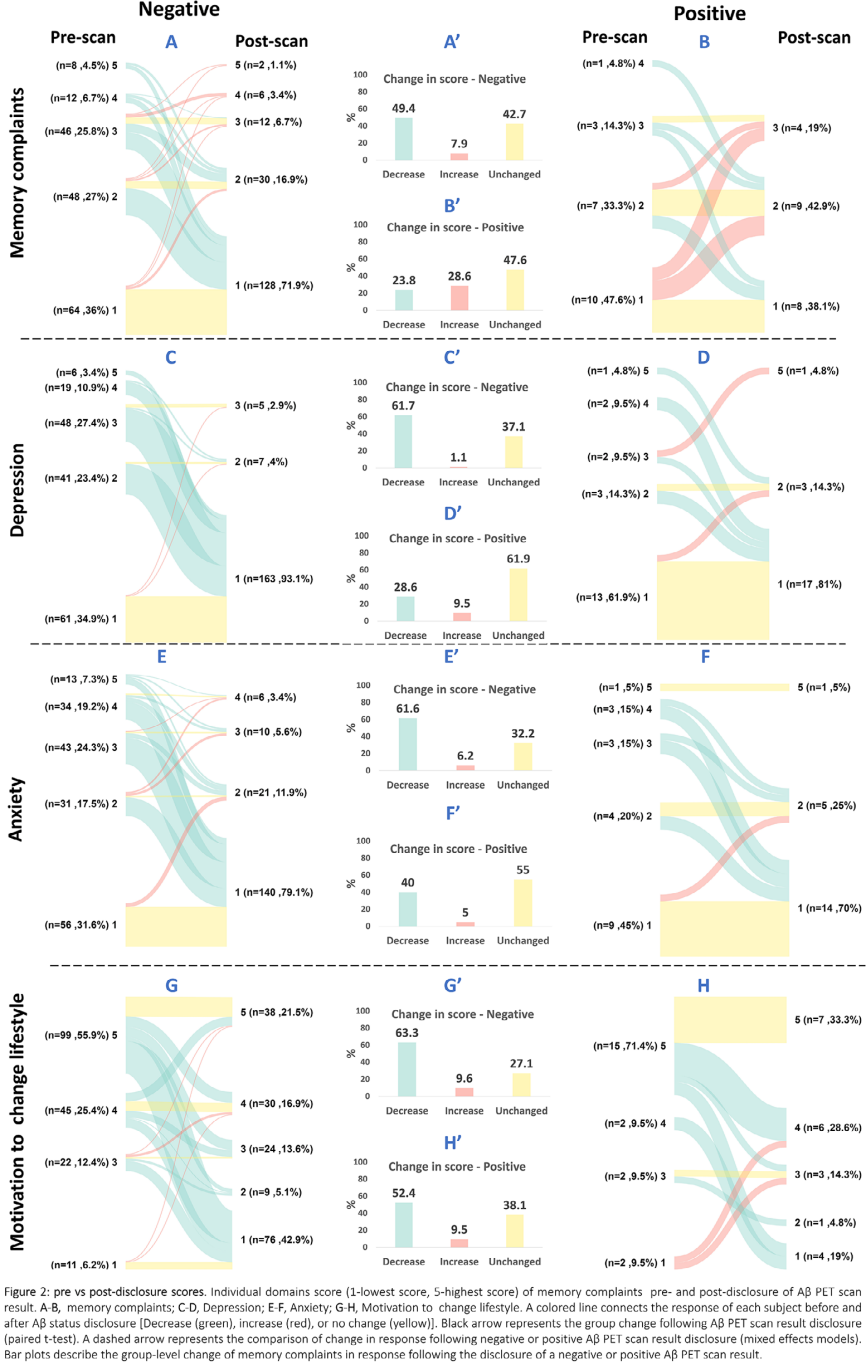# Behavioral Reaction to Amyloid β Status Disclosure Among Research Participants with High‐AD Risk in Israel

**DOI:** 10.1002/alz.088020

**Published:** 2025-01-09

**Authors:** Sapir Golan, Michal S Beeri, Maya Zadok, Mery Ben‐Meir, Yael Rozen, Revital Shutsberg, Ariela Bem‐Moshe, Anthony Heymann, Yossi Azuri, Ithamar Ganmore, Ramit Ravona‐Springer, Chen Hoffmann, Liran Domachevsky, Orit H. Lesman‐Segev

**Affiliations:** ^1^ Sackler Faculty of Medicine, Tel Aviv University, Tel Aviv Israel; ^2^ The Joseph Sagol Neuroscience Center, Sheba Medical Center, Tel Hashomer Israel; ^3^ Krieger Klein Alzheimer's Research Center, New Brunswick, NJ USA; ^4^ Herbert and Jacqueline Krieger Klein Alzheimer’s Research Center at Rutgers Brain Health Institute, New Brunswick, NJ USA; ^5^ Maccabi Health Services, Tel Aviv Israel; ^6^ Memory clinic, Sheba Medical Center, Tel Hashomer Israel; ^7^ Faculty of Medical and Health Science, Tel Aviv University, Tel Aviv Israel; ^8^ Department of Diagnostic Imaging, Sheba Medical Center, Tel Hashomer Israel

## Abstract

**Background:**

Amyloid beta (Aβ) deposition marks an early stage in the progression of Alzheimer's disease (AD), detectable in‐vivo years before symptoms emerge and targeted by recently FDA‐approved drugs. This has propelled advancements in understanding, measuring, and treating AD, paving the way for disease prevention in those at risk. However, the psychological impact of disclosing Aβ status to cognitively unimpaired individuals remains underexplored. Our study aimed to evaluate the behavioral responses to Aβ status disclosure in this population.

**Method:**

Two observational studies and two clinical trials incorporating Aβ‐PET were conducted, involving research participants who received information on Aβ‐PET scans, results, and interpretation. Questionnaires were administered before the scan and four‐to‐six months post‐disclosure to assess anxiety, depression, subjective memory complaints, and motivation for risk‐reduction behaviors. Bivariate analysis and mixed models were employed to analyze responses, with logistic regression used to identify predictors of unfavorable responses to negative scan disclosure.

**Result:**

Among 178 participants with amyloid‐negative scans (mean age 64, 57% females) and 21 with amyloid‐positive scans (mean age 77, 38% females), no significant pre‐scan differences were observed. Negative amyloid disclosure correlated with reductions in all domains tested, including memory complaints, depression, anxiety, and motivation to change lifestyle (p≤0.001). Positive amyloid disclosure also resulted in decreases, particularly in anxiety and depression, albeit to a lesser extent and with greater variability (0.007<p<0.07 for pre to post PET change in amyloid positives vs. negatives). Higher education was linked to fewer memory complaints post‐negative Aβ PET disclosure (Odds ratio=0.79, CI=0.65‐0.97, p=0.02).

**Conclusion:**

In conclusion, as AD enters an era of disease‐modifying and preventive treatments, understanding how cognitively normal adults respond to AD biomarker status disclosure becomes crucial. Negative amyloid results offer a comforting effect, reflected in reduced subjective memory concerns and lower anxiety, depression, and motivation for lifestyle changes. However, caution is needed to prevent false reassurance. Positive amyloid results also provide comfort, albeit to a lesser extent and with more variability. Lower education levels may predict unfavorable responses to negative amyloid disclosures.